# Prevalence and antimicrobial resistance pattern of Shiga toxigenic *Escherichia coli* in diarrheic buffalo calves

**DOI:** 10.14202/vetworld.2017.774-778

**Published:** 2017-07-13

**Authors:** M. Srivani, Y. Narasimha Reddy, K. V. Subramanyam, M. Ramakoti Reddy, T. Srinivasa Rao

**Affiliations:** 1 Department of Veterinary Microbiology, NTR College of Veterinary Science, Gannavaram, Krishna - 521 101, Andhra Pradesh, India; 2 Department of Veterinary Microbiology, College of Veterinary Science, Rajendranagar, Hyderabad, Telangana, India; 3 Directorate of Poultry Research, Rajendranagar, Hyderabad, Telangana, India; 4 Department of Veterinary Public Health, NTR College of Veterinary Science, Gannavaram, Andhra Pradesh, India

**Keywords:** antimicrobial resistance, buffalo calf diarrheia, Shiga toxigenic *Escherichia coli*, virulence genes

## Abstract

**Aim:**

Aim of the study was to investigate the prevalence, virulence gene profiles, and antimicrobial resistance pattern of Shiga toxigenic *Escherichia coli* (STEC) in diarrheic buffalo calves from Andhra Pradesh and Telangana States.

**Materials and Methods:**

A total of 375 fecal samples from diarrheic buffalo calves of 1-7, 8-30, 31-60, and 61-90 days age were collected from which STEC were isolated, and virulence genes were detected using multiplex polymerase chain reaction. The antimicrobial resistance of isolates was tested by disk diffusion method.

**Results:**

The prevalence of *E. coli* associated diarrhea in buffalo calves was 85.04%, of which 35.01% was STEC origin. In STEC, the combination of *eaeA* and, *hlyA* virulence genes was highest (42.45%) followed by *stx1* (16.04%), *stx1, stx2* and *hlyA* (13.21%), *stx2* (12.64%), *stx1, eae* and *hlyA* (9.43%) and *stx1* and *hlyA* (6.6%) genes were detected. Highest antimicrobial resistance was observed for tetracycline (63.21%) and ampicillin (48.11%), while chloramphenicol, gentamycin (96.33%) and imipenem (99.06%) antibiotics are susceptible. Multidrug resistance was detected in 69.81% of the STEC isolates from diarrheic buffalo calves.

**Conclusion:**

Higher prevalence of *eaeA* and *hlyA* genes carrying isolates of STEC may be a serious zoonotic threat and increased prevalence of multidrug resistance in *E. coli* may necessitate stringent selection of appropriate antimicrobial agent in treating buffalo calf diarrhea cases.

## Introduction

Calf diarrhea is a complex syndrome with complex etiopathogenesis causing economic loss directly through mortality and indirectly through treatment costs and reduced growth rates in affected calves [1]. The mortality rate is high, particularly in buffalo calves of <3 months age in India [2]. Among all the etiological agents responsible for calf diarrhea, *Escherichia coli* are recognized as the leading cause [3]. Shiga toxigenic *E. coli* (STEC) is one of the pathogenic groups of *E. coli* that has zoonotic origin and cattle being recognized as the major reservoir for human infections.

In calves, STEC is the main cause of diarrhea and dysentery particularly in very young calves [4]. The pathogenicity of STEC is mediated mainly through Shiga toxins 1 and 2 encoded by *stx1* and *stx2* genes, respectively [5], intimin, an outer membrane surface adhesin encoded by *eaeA* gene [6] and enterohemolysin (*ehly*) which is encoded by the *hlyA* gene [7]. Antimicrobial therapy is an important tool in reducing the incidence and treatment of diarrhea in calves. However, widespread and indiscriminate use of antimicrobial agents leads to multi-drug resistant pathogenic bacteria in calves, resulting difficulty in treatment [8].

Therefore, the aim of this study was to investigate the diversity and distribution of virulence genes and to understand antimicrobial resistance epidemiology of STEC isolated from diarrheic buffalo calves in Andhra Pradesh and Telangana States.

## Materials and Methods

### Ethical approval

Ethical approval was not necessary for this study. However, samples were collected as per standard collection procedure without harming or giving stress to the animals.

### Sample collection

A total of 375 fecal samples from diarrheic buffalo calves of 1-7, 8-30, 31-60, and 61-90 days age were collected randomly from organized dairy farms and individual farmers of Vizianagaram, Vishakapatnam, East Godavari, West Godavari, Krishna, Guntur, Prakasam, Districts of Andhra Pradesh State and Ranga Reddy and Khammam Districts of Telangana State during the period from May 2014 to November 2015. Geographical distribution and age of diarrheic calves were recorded during sampling. Fecal samples were collected using sterile rectal swabs. After collection, the swabs were immediately transported to the Department of Veterinary Microbiology, NTR College of Veterinary Science, Gannavaram in ice-cooled containers for *E. coli* isolation. All the samples were inoculated on MacConkey agar and incubated at 37°C for 24 h. The pink colonies obtained were again inoculated on EMB agar, and the colonies showing green metallic sheen were selected and confirmed as *E. coli* by standard biochemical tests [9] and by polymerase chain reaction (PCR) amplifying 16S rRNA gene [10].

### DNA isolation

Isolation of DNA from *E. coli* was carried out by conventional boiling and snap chilling method [11] with slight modifications. A single colony was inoculated in 1 ml tryptic soy broth and incubated at 37°C for 24 h. The cells were harvested by centrifugation at 5000 rpm for 10 min. The pellet was washed with phosphate buffer saline by centrifuging at 500 rpm for 10 min for twice. Then, the pellet was resuspended in 500 µl nuclease free water and boiled for 5-10 min at 100°C and snap chilled on ice, after centrifugation at 1000 rpm for 5 min; supernatant was used as template DNA.

### Detection of Shiga toxigenic E. coli

The primers used in the present study for the detection of Shiga toxin producing *E. coli* were as described [5] (Table-1). Multiplex PCR for amplification of the *stx*1, *stx*2, *eaeA*, and *hlyA* genes was set up in 25 µl reaction contained 3 µl of bacterial lysate, 2.5 µl of 10× PCR buffer with 1.5 mM of MgCl_2_, 250 nM of each forward primer and reverse primer, 0.5 µl of 10 Mm dNTP’S and 0.5 units of Taq DNA polymerase, and autoclaved milli-Q water to make volume to 25 µl. Samples were subjected to 35 PCR cycles, each consisting of 1 min of denaturation at 95°C; 2 min of annealing at 65°C for the first 10 cycles, decrementing to 60°C by cycle 15, and 1.5 min of elongation at 72°C, incrementing to 2.5 min from cycles 25 to 35. PCR reaction mixtures were electrophoresed on 2% agarose gels and stained with ethidium bromide.

**Table-1:** Details of the primers used for the detection of *stχ*1, *stχ*2, *eae*A and *hly* A genes.

S. No	Primer	Sequence (5’---- 3’)	Target gene	Expected amplicon size (bp)
1	*Stχ*1F	ATAAATCGCCATTCGTTGACTAC	*stχ*1	180
	*Stχ*2 R	AGAACGCCCACTGAGATCATC		
2	*Stχ*2 F	GGCACTGTCTGAAACTGCTCC	*stχ*2	254
	*Stχ*2 R	TCGCCAGTTATCTGACATTCTG		
3	*eaeA* F	GACCCGGCACAAGCATAAGC	*eae*A	384
	*eaeA* R	CCACCTGCAGCAACAAGAGG		
4	*hlyA* F	GCATCATCAAGCGTACGTTCC	*hly*A	534
	*hlyA* R	AATGAGCCAAGCTGGTTAAGCT		

### Antibiotic susceptibility testing

Antimicrobial resistance against 18 different antibiotics, i.e., for ampicillin, cefotaxime, ceftazidime, amoxicillin clavulanic acid, gentamycin, kanamycin, streptomycin, sulfisoxazole, cotrimoxazole, nalidixic acid, ciprofloxacin, aztreonam piperacillintazobactem, tetracyclin, chloramphenicol, meropenem, imipenem, and nitrofurantoin was studied by disk diffusion method [12]. The diameter of the zone of inhibition was compared with the standard known value against each specific antimicrobial agent as suggested in the product information (interpretation guideline) from manufacturer.

## Results and Discussion

This study detected 80.53% prevalence of *E. coli* in diarrheic buffalo calves in AP and TS states (Table-2), and the prevalence (88.20%) was highest in commercial diaries than in buffalo calves maintained by individual farmers (72.86%). This might be due to unhygienic housing, poor feeding, and management of buffalo calves in commercial diaries than at individual farmers. The prevalence rate reported in this study was higher than prevalence of *E. coli* reported in diarrheic buffalo calves in Jabalpur, India (59.37%) [13], Egypt (66 and 72%) [14,15], Pakistan (14.6%) [16], and Bangladesh (45) [17]. The differences of the prevalence rates of *E. coli* in diarrheic calves may be attributed to the geographical locations and management practices as well as hygienic measures which influence the susceptibility of calves to *E. coli* infection [18,19].

**Table-2:** Prevalence of *E. coli* in the fecal samples of diarrheic calves obtained from different ages.

Age (days)	Number of samples collected	Number positive for *E. coli*	Prevalence (%)
1-7	127	108	85.04
8-30	187	157	83.96
31-60	49	31	63.27
61-90	12	6	50.00
Total	375	302	80.53

E. coli: Escherichia coli

This study also observed the highest prevalence of *E. coli* (85.04%) associated diarrhea in 1-7 days age buffalo calves followed by 83.96% and 63.27% prevalence in 8-30 and 31-60 day age groups, while lowest (50.0%) prevalence was observed in 61-90 days age buffalo calves (Table-2). Higher prevalence in younger calves may be due to increased susceptibility to *E. coli* infection [20-22] and predisposing factors like overcrowding and malnutrition, which are supposed to be a primary cause of immunosuppression [14]. Further, *E. coli* is a commensal organism and is responsible for diarrhea in calves, particularly calves receiving less or no maternal antibodies through colostrum where milk is mainly used for commercial purposes [23].

**Figure-1:**
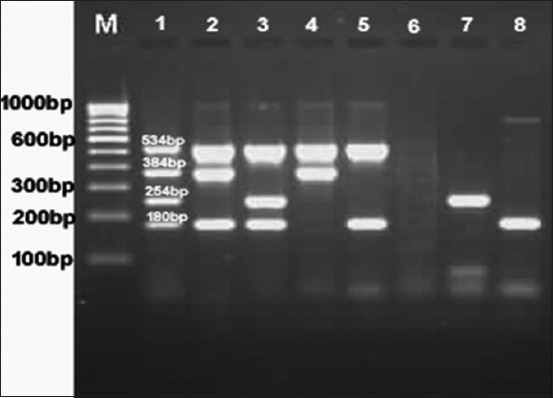
Multiplex polymerase chain reaction detecting the virulence genes. Lane M: 100 bp DNA ladder, Lane 1: Standard *stx*1 (180 bp), *stx*2 (254 bp), *eae*A (384 bp), *hlyA* (534 bp), Lane 6: Negative, Lane 2-5,7 and 8: *Escherichia coli* isolates from diarrheic buffalo calves.

Among the *E. coli* isolates, 35.10% were detected as STEC by multiplex PCR (Figure-1) which was higher (6.8%) and lower (47.30%) than STEC reported from fecal samples of diarrheic buffalo calves in Italy [24] and diarrheic calves in Iran [25]. Studies carried out in different countries by several researchers revealed that 10-80% of cattle may carry STEC [26]. These differences might be due to variation in environment which has an influence on the shedding of STEC in calves [27].

The virulence gene profile of the STEC isolates from diarrheic calves was found in diverse combinations (Table-3 and Figure-1).

**Table-3:** Distribution of virulence genes among STEC isolates in diarrheic buffalo calves.

Total *E. coli* isolates	STEC	Virulence gene	Number of isolates	% in STEC
302	106 (35.10%)	*Stχ1*	17	16.04
		*Stχ2*	13	12.26
		*Stχ1stχ2HlyA*	14	13.21
		*Stχ1EaeAHlyA*	10	9.43
		*Stχ1HlyA*	7	6.60
		*EaeA, HlyA*	45	42.45

*E. coli: Escherichia coli*, STEC: Shiga toxigenic *Escherichia coli*

The STEC isolates carrying *eaeA* and *hlyA* genes were most prevalent (42.45%). Similar prevalence rate of *eaeA* and *hlyA* genes harboring isolates from diarrheic calves was also reported in other studies [6,28]. Several investigators have reported that the strong association between the carriage of *eaeA* gene, and the capacity of STEC to cause severe human disease [6,29]. Therefore, higher prevalence of *eaeA* and *hlyA* genes in STEC isolates from diarrheic buffalo calves detected in the present study may be a serious zoonotic threat in this geographic region. The STEC isolates carrying 16.04% of *Stx1* and 12.26% of *Stx2* genes in the present study was lower than reported in Iran [30] but higher than reported in India [31], Poland [32], and Turkey [33]. However, higher prevalence of *stx1* gene than *stx2* gene observed from diarrheic calves in the present study is comparable to the observations in Argentina [34] and in Austria [35].

The antimicrobial susceptibility testing revealed 69.81% of the STEC isolates were resistant to three or more of the antimicrobial agents tested. Among the STEC isolates, highest percentage of antimicrobial resistance was observed for tetracycline (63.21), followed by ampicillin (48.11%), aztreonam (36.79%), cefotaxime, ceftazidime, and streptomycin, (31.13%), nalidixic acid (29.25%), sulfisoxazole (28.30%), cotrimoxazole (26.42%), amoxicillin clavulanic acid (20.75%), piperacillintazobactem (18.87%) meropenem (17.92%) kanamycin and nitrofurantoin (12.26%) ciprofloxacin (4.72), chloramphenicol and gentamycin (3.77%) while lowest % of 0.94 was observed for imipenem antibiotics. The present findings were corroborated with findings of 100% multidrug resistance in STEC isolates from diarrheic calves in Brazil [36].

Several investigators [36-38] around the world also detected highest antibiotic resistance for *E. coli* isolates from diarrheic calves to tetracycline antibiotic. Highest sensitivity of the STEC isolates to chloramphenicol and gentamycin antibiotics observed in the present study was comparable with findings of Wani *et al*. [31] and Rehman *et al*. [39] who reported higher sensitivity for STEC isolates from diarrheic calves to chloramphenicol and gentamycin antibiotics in Jammu and Kashmir of India.

The present results concluded higher prevalence of *eaeA* and *hlyA* genes in STEC from diarrheic buffalo calves may be a serious zoonotic threat in this geographical region. Further, the multidrug resistance of STEC isolates may necessitates stringent selection of appropriate antimicrobial agent and judicious use in treating buffalo calf diarrhea cases.

## Authors’ Contributions

MS carried out the research work. YNR and KVS designed and supervised the experiment. TSR helped in carried out PCR analysis. MRR did the data analysis. All authors read and approved the final manuscript.
